# Hypertensive Urgency and Pulmonary Infiltrates: A Report of Three Cases

**DOI:** 10.1002/ccr3.72429

**Published:** 2026-04-09

**Authors:** Mónica Contreras‐Moreira

**Affiliations:** ^1^ Community of Madrid Centro de salud “La Ventilla” Madrid Spain

**Keywords:** asymptomatic, hypertensive crisis, pulmonary infiltrates, ultrasound

## Abstract

Hypertensive urgency is commonly attributed to medication nonadherence, progression of essential hypertension, or secondary causes. However, in some patients, no clear precipitating factor is identified, despite repeated evaluations and the absence of target organ damage. Subclinical inflammatory conditions have been hypothesized as potential, underrecognized contributors to acute blood pressure destabilization. We describe three cases of elderly women with previously diagnosed and treated essential hypertension who presented with episodes of hypertensive urgency without evidence of acute target organ damage. In each case, blood pressure remained persistently elevated despite optimization of oral antihypertensive therapy and exclusion of common secondary causes. Lung ultrasound performed during evaluation revealed pulmonary parenchymal infiltrates suggestive of an inflammatory process, despite minimal or absent respiratory symptoms and unremarkable laboratory tests, electrocardiograms, and chest radiographs. Although microbiological confirmation and inflammatory biomarker elevation were not documented, empirical antibiotic therapy was initiated based on clinical judgment. Follow‐up lung ultrasound demonstrated progressive improvement of pulmonary findings, temporally associated with stabilization and improved blood pressure control, allowing simplification or return to baseline antihypertensive regimens. These cases suggest a temporal association between lung ultrasound–detected pulmonary infiltrates and hypertensive urgency in patients with essential hypertension. While causality cannot be established, subclinical inflammatory pulmonary processes may represent potential contributors to acute blood pressure elevation in some patients. Lung ultrasound may serve as a useful adjunctive tool in the evaluation of unexplained hypertensive urgency. Prospective studies incorporating microbiological and inflammatory markers are needed to further clarify this possible association.

## Graphical abstract

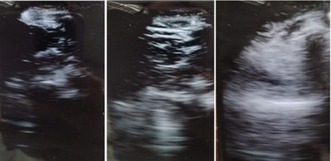

**Key Clinical Message**

Hypertensive urgency without target organ damage may be associated with occult pulmonary infiltrates. In selected asymptomatic patients with unexplained elevated blood pressure, lung ultrasound may help identify subclinical pulmonary infiltrates. Although causality cannot be established, the observed temporal association between empirical treatment and blood pressure improvement requires further investigation.

## 1 Introduction

Hypertensive crises are a frequent cause of emergency and urgent care visits among patients with known hypertension. Many episodes are often related to medication nonadherence, disease progression, or identifiable secondary causes. However, some patients experience recurrent hypertensive urgencies without clear precipitating factors or evidence of acute target organ damage. Identifying reversible triggers in these situations is clinically relevant, as it may prevent repeated healthcare utilization and unnecessary escalation of antihypertensive therapy.

Arterial hypertension or high blood pressure (HBP) is defined as a sustained elevation in blood pressure, with essential, primary, or idiopathic hypertension affecting 80%–95% of hypertensive patients. Secondary hypertension is diagnosed when a specific underlying factor is identified as causing increased blood pressure [1].

Besides cardiac output and peripheral vascular resistance as primary determinants of blood pressure, its regulation is a complex, multifactorial process involving intravascular volume homeostasis, the renin–angiotensin–aldosterone system, the autonomic nervous system, endothelial dysfunction, disturbances in sodium handling, immunological pathways, insulin resistance, obesity, environmental and behavioral factors, and genetic susceptibility [1, 2, 3, 4].

Secondary hypertension can be caused by renal parenchymal disease, renovascular diseases, primary aldosteronism, Cushing's syndrome, pheochromocytoma, obstructive sleep apnea, aortic coarctation, thyroid disorders, acromegaly, hypercalcemia, and drug use [1]. Less common causes include pregnancy, renin‐secreting tumors, hyperparathyroidism, central nervous system disorders, blood hyperviscosity, and other rare conditions [5]. In patients diagnosed with essential hypertension, even without identifiable secondary causes, the determinants already mentioned [2, 3] can precipitate hypertensive crises, defined as acute elevations in blood pressure, above 180 and/or 120 mmHg for systolic and diastolic values, respectively.

Community‐acquired pneumonia (CAP) often presents with nonspecific symptoms, though its typical features include acute fever > 38°C, purulent productive cough, pleuritic chest pain, and dyspnea. Atypical presentations may be subacute, presenting with dry cough, gastrointestinal symptoms in up to 20% of cases, and, in the case of older adults, no fever but confusion or worsening of comorbidities. No single history or physical examination finding is sufficient for diagnosis, and clinical prediction rules have therefore been developed.

Diagnosis of CAP relies on the combination of clinical presentation and radiographic findings. When clinical suspicion is high despite negative radiographs, empirical antibiotic therapy should be initiated promptly [6, 7, 8].

On the other hand, it is known that respiratory infections induce systemic inflammation, endothelial dysfunction, and sympathetic activation [9, 10, 11], all of which may influence blood pressure regulation. However, the role of asymptomatic pulmonary infections associated with hypertensive urgency has been poorly explored. Most diagnostic algorithms for hypertensive crises do not include evaluation for occult infection in the absence of clinical or laboratory signs. This gap may lead to underdiagnosis of reversible contributors to blood pressure destabilization.

Lung ultrasound has proven to be a sensitive bedside technique capable of detecting pulmonary infiltrates and interstitial syndrome even in the absence of respiratory symptoms or radiographic abnormalities [12, 13, 14]. Unlike chest x‐ray, which has limited sensitivity for small or posterior consolidations, lung ultrasound (LUS) can effectively visualize early changes such as focal B‐lines and subpleural consolidations, allowing for the detection of peripheral lesions and silent progression in patients before the onset of severe respiratory symptoms [15, 16, 17]. High sensitivity and specificity were observed for LUS in diagnosing CAP, by showing infiltrates such as air bronchograms and pleural involvement, even when radiographs were inconclusive [18, 19].

Current international clinical guidelines support the early initiation of empirical antibiotic therapy in patients with suspected CAP, and it is recommended to start antibiotics based on clinical presentation and the expected prevalence of common pathogens, as delayed treatment has been associated with worse outcomes [6]. Spanish primary care guidelines emphasize that lower respiratory tract infections are frequent in the Primary Care setting and often justify early empirical treatment [6, 7, 8].

In accordance with the CARE guidelines for case reports [20], we describe three cases of women with treated essential hypertension who presented with hypertensive urgency, in whom lung ultrasound revealed pulmonary infiltrates during evaluation.

## 2 Case Series

A comparative summary of the three cases, including timelines, diagnostic findings, treatments, and outcomes, is provided in Table 1.

**TABLE 1:** Patient's management.

Clinical moment	Patient 1 (≈14 visits)	Patient 2 (4 visits)	Patient 3 (3 visits)
Initial hypertensive crisis	01/22: BP 200/120, poor general condition. In hospital: ECG, labs and CXR normal. Dx: resolved hypertensive crisis.	03/17: BP 200/90, headache and dizziness. After captopril, BP 190/93 → referred to ER.	05/21: reported BP ~200/100 during trip, dizziness. At visit: 161/92, O2 sat 94%, normal ECG.
Hospital/ER evaluations	Several (01/22, 01/29, 02/10, 03/21): normal exams and tests; repeated Dx of resolved hypertensive crisis.	03/17 (later): BP 183/128, HR 114, O2 sat 96%, normal exam and labs. Dx: resolved hypertensive urgency.	—
Pulmonary infiltrate finding	01/27: left basal infiltrate (US). 03/11: extensive left basal infiltrate.	03/24: diffuse bilateral infiltrates (US).	05/21: extensive bibasal infiltrates (US).
Initial antibiotic treatment	Amoxicillin 500 mg q8h for 6 days (01/27). Later cefditoren 400 mg + acetylcysteine 10 days (03/11).	Amoxicillin 750 mg q8h for 6 days (03/24).	Amoxicillin 1,000 mg q8h for 6 days + acetylcysteine (05/21).
Follow‐up and BP control	Multiple visits (Feb–Jun): adjustments of enalapril/HCTZ ± amlodipine, palpitations, anxiety. BP stabilized.	04/02: home BP ~140/65 on lercanidipine + doxazosin.	06/02: home BP 150/80, then 140/70–143/58 on telmisartan.
Persistence/evolution of infiltrates	03/24 and 04/22: infiltrates improving, residuals. 06/09: asymptomatic, residual infiltrate.	04/02: clinical improvement, infiltrates resolving.	06/02 and 06/16: infiltrates still present, smaller extent.
Final follow‐up treatment (vs. initial)	Final: ACEi + HCTZ ± amlodipine, acetylcysteine as needed. Initial: enalapril only, no HCTZ or amlodipine.	Final: lercanidipine + doxazosin. Initial: captopril (single dose in crisis), later amlodipine.	Final: losartan/HCTZ + acetylcysteine. Initial: high‐dose telmisartan, no diuretic.

### 2.1 Case 1

#### 2.1.1 Patient Information

A 70‐year‐old woman with a history of essential hypertension, lymphocytic colitis, recurrent renal colic (asymptomatic at presentation), and appendectomy. Chronic treatment included enalapril 10 mg daily.

#### 2.1.2 Presenting Concerns

In January 2022, she presented with a hypertensive crisis (BP 200/120 mmHg) associated with malaise, motion sickness, and blurred vision, prompting referral to the emergency department.

#### 2.1.3 Clinical Findings

Physical examination and neurological assessment were unremarkable. No respiratory symptoms were reported.

#### 2.1.4 Diagnostic Assessment

**FIGURE 1:**
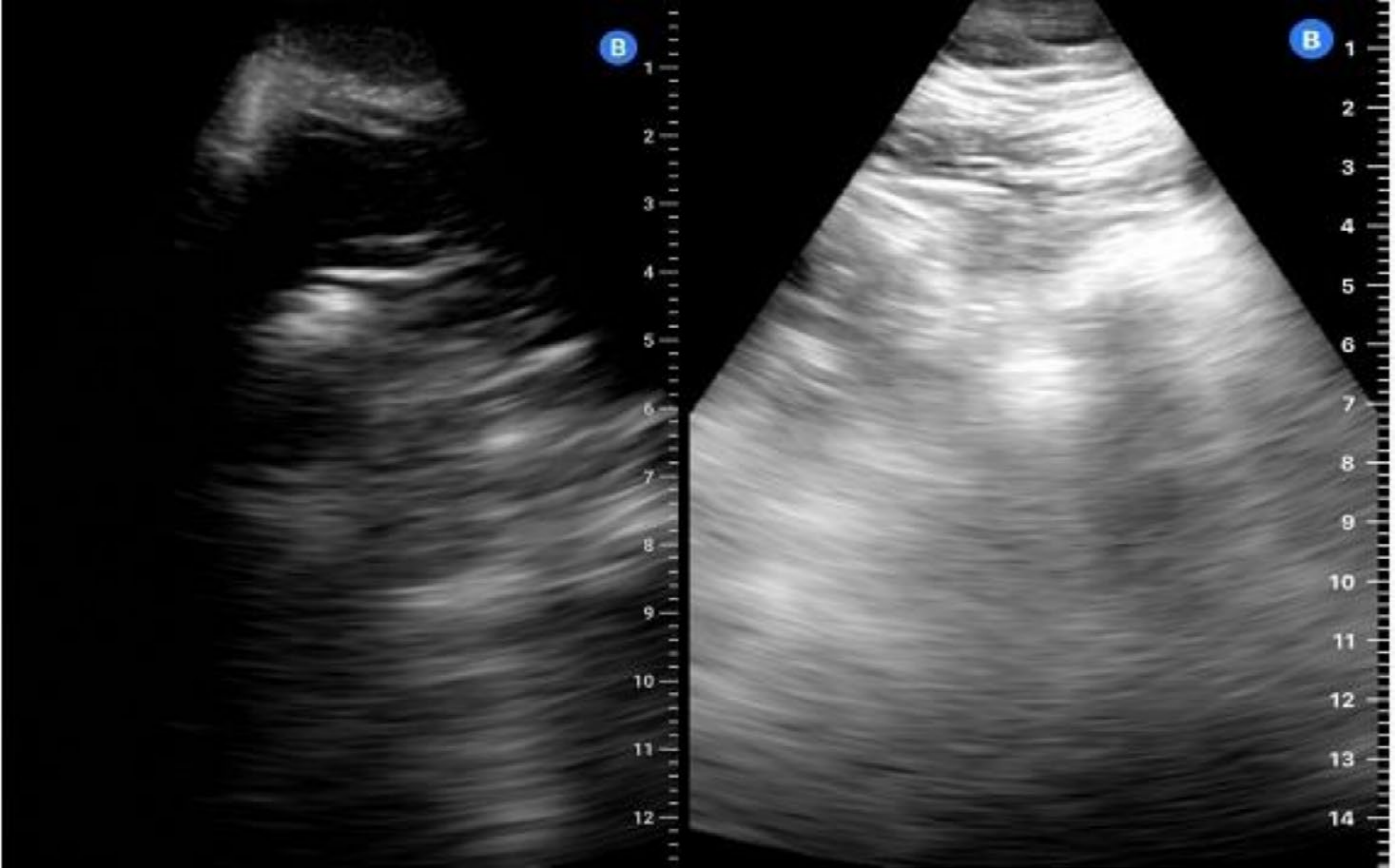
Patient 1. Ultrasound performed with the portable “Butterfly” device of the left lung base in posterior view, showing a small subpleural and intraparenchymal infiltrate (left lateral on the left) and an increase in consolidation foci and loss of the A‐line pattern (left medial on the right).

Electrocardiogram, laboratory studies, and chest X‐ray showed no evidence of acute target organ damage. The episode was classified as hypertensive urgency. Differential diagnoses considered included medication nonadherence, secondary hypertension, anxiety‐related blood pressure elevation, and occult target organ damage. These were ruled out based on the patient's interview and the clinical assessment. Due to persistent blood pressure instability and nonspecific symptoms over subsequent visits, a lung ultrasound was performed. It revealed a left basal intraparenchymal infiltrate, which progressed on the follow‐up examination (Figure 1). Differential diagnosis for the hypertensive crises included poor therapeutic adherence, the effect of NSAIDs, decongestants, corticosteroids, or abused substances, acute pain or anxiety, all of which had been ruled out during the clinical interview. Acute renal failure, given the preserved diuresis, was also discarded.

Pulmonary edema was thought of as a differential diagnosis, but was rejected due to the basal‐only location of the infiltrates and the absence of generalized B‐lines in LUS.

#### 2.1.5 Therapeutic Intervention

Antihypertensive therapy was optimized with enalapril and hydrochlorothiazide, with subsequent addition of amlodipine as needed. The identification of parenchymal infiltrates on lung ultrasound examination and the presence of malaise, HBP, motion sickness, and blurred visión, despite the absence of overt respiratory symptoms, led to the decision to initiate empirical antibiotic therapy [6, 7, 8]. It started with amoxicillin [21], followed by cefditoren [22] plus acetylcysteine [22, 23] due to persistent ultrasound findings on the following examination.

#### 2.1.6 Follow‐Up and Outcomes

Blood pressure gradually stabilized over subsequent visits. Lung ultrasound showed progressive improvement, with residual findings at three months. The patient became asymptomatic with blood pressure within the target range.

### 2.2 Case 2

#### 2.2.1 Patient Information

A 75‐year‐old woman with essential hypertension, type II diabetes mellitus, dyslipidemia, and hyperuricemia. Chronic medications included lercanidipine, doxazosin, metformin/sitagliptin, dapagliflozin, repaglinide, simvastatin, and allopurinol.

#### 2.2.2 Presenting Concerns

She presented in March 2025 with insomnia, dizziness, and headache. Repeated blood pressure measurements showed an average of 200/90 mmHg.

#### 2.2.3 Clinical Findings

Vital signs were otherwise stable. No respiratory symptoms were reported.

#### 2.2.4 Diagnostic Assessment

Laboratory tests and ECG remained normal. Despite oral antihypertensive treatment, blood pressure remained elevated. Differential diagnosis included physiological stress, which was rejected during the clinical interview, and hydrosaline retention, which seemed unlikely given the absence of edema and the preserved diuresis.

**FIGURE 2:**
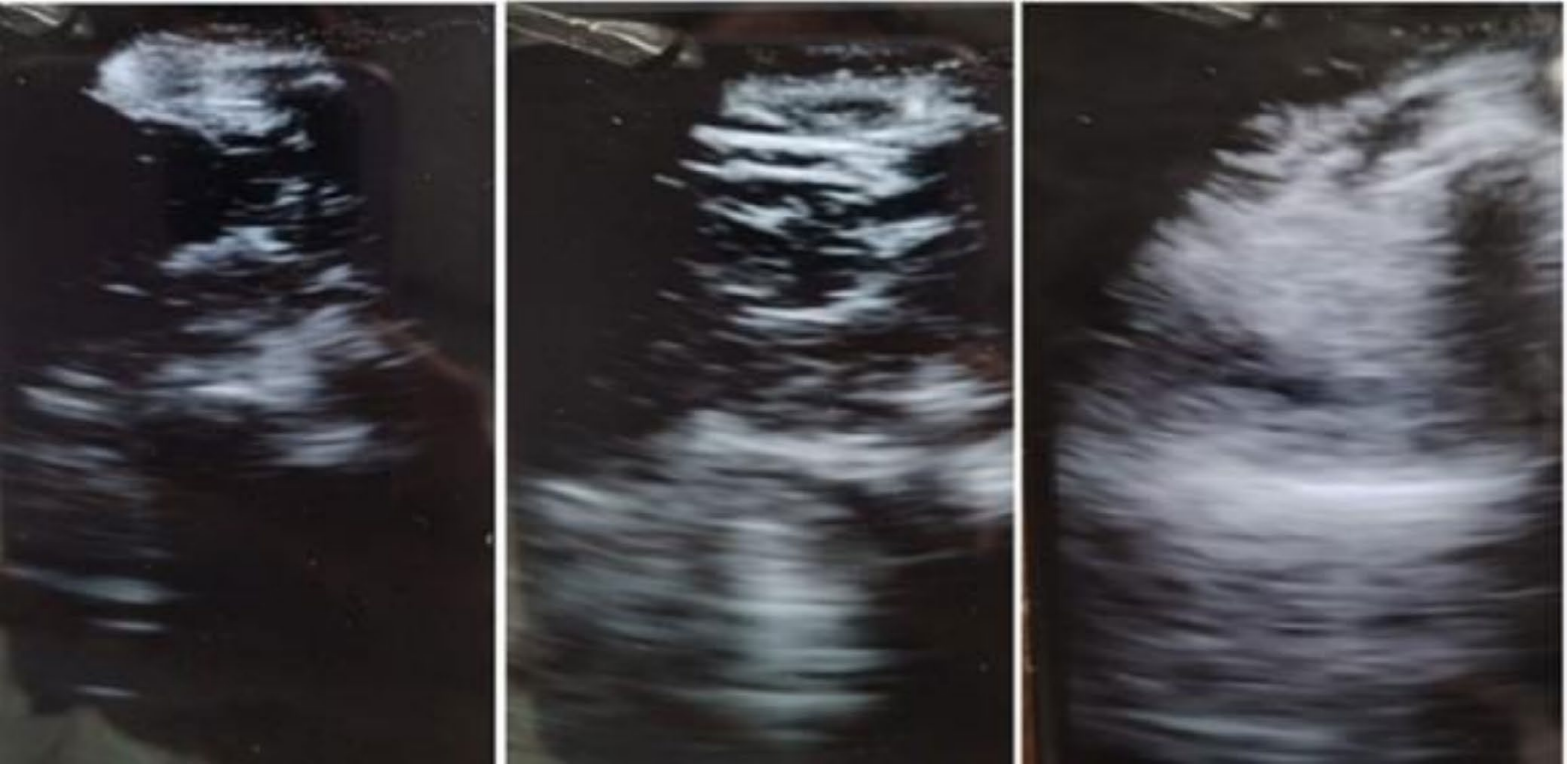
Patient 2. Left and center: Ultrasound of the left lung base in posterior view, showing several foci of intraparenchymal infiltrates. Right: Anterior fields of the right lung base, showing a large intraparenchymal infiltrate.

As no trigger was identified, a lung ultrasound was performed, showing diffuse bilateral intraparenchymal infiltrates (Figure 2). Interstitial involvement or pulmonary congestion was ruled out in LUS, favoring an inflammatory etiology over congestive causes.

#### 2.2.5 Therapeutic Intervention

Empiric antibiotic treatment was initiated, given the presence of ultrasound‐confirmed pulmonary infiltrates and the persistence of HBP and nonspecific symptoms (insomnia, dizziness, headache). Amoxicillin [21] was prescribed.

#### 2.2.6 Follow‐Up and Outcomes

Follow‐up ultrasound showed progressive resolution of infiltrates, and blood pressure normalized, allowing return to baseline antihypertensive therapy.

### 2.3 Case 3

#### 2.3.1 Patient Information

A 78‐year‐old woman with essential hypertension treated with telmisartan 20 mg daily.

#### 2.3.2 Presenting Concerns

She reported home blood pressure readings near 200/100 mmHg during travel, with occasional dizziness.

#### 2.3.3 Clinical Findings

At evaluation, repeated blood pressure measurements showed an average of 161/92 mmHg, and oxygen saturation was 93%. No respiratory symptoms were present.

#### 2.3.4 Diagnostic Assessment

**FIGURE 3:**
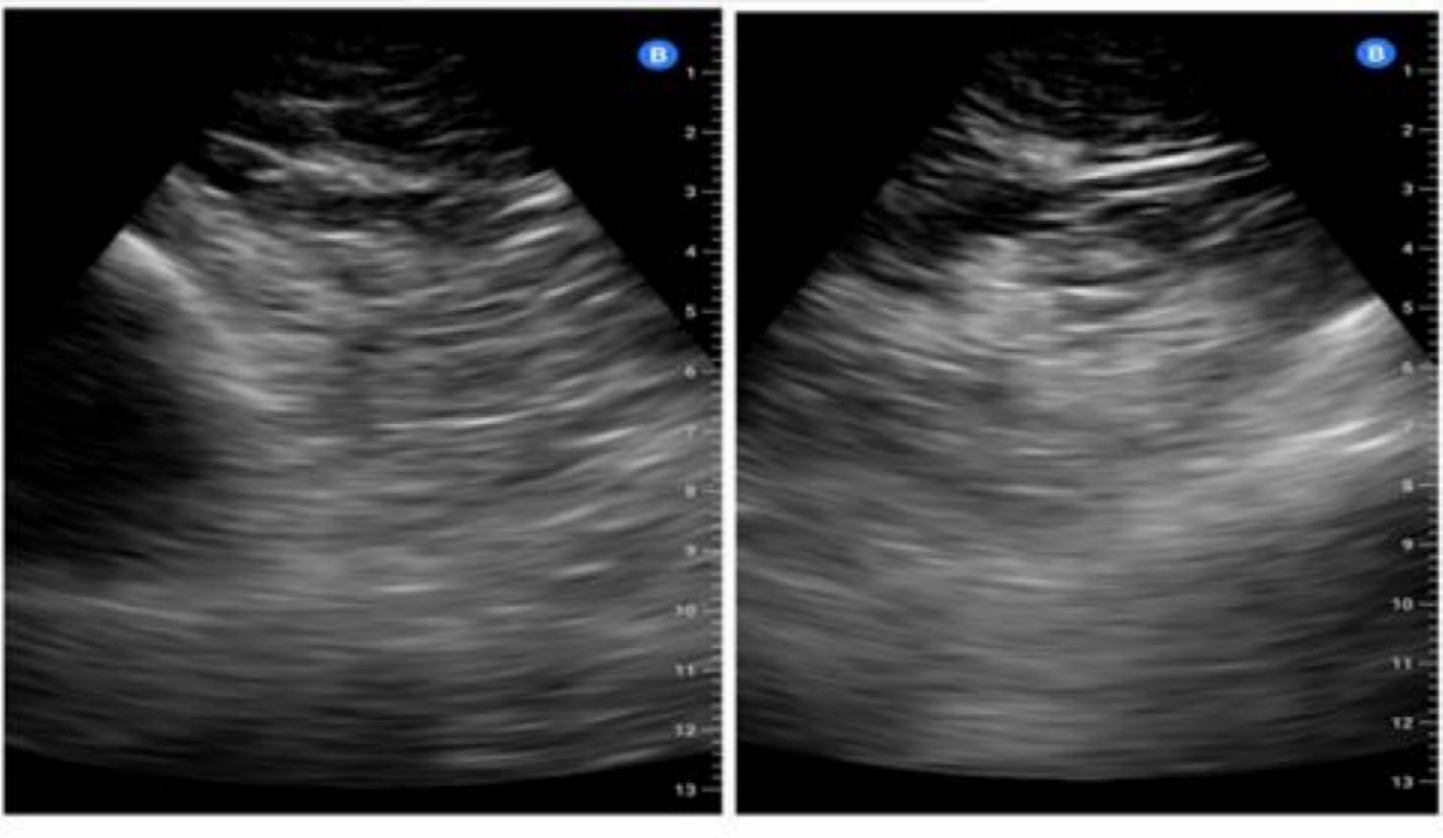
Patient 3. Ultrasound of the left and right lung bases, respectively, in posterior view, showing small foci of fluid bronchograms scattered throughout the parenchyma, slightly more organized in the right lung.

ECG and laboratory studies were normal. Given the low oxygen saturation, a lung ultrasound was performed, revealing extensive bilateral basal infiltrates within the lung parenchyma (Figure 3). The rapid onset of high figures made it difficult to consider it as the natural progression of the disease; a drug interaction was suspected during the clinical interview; hydrosaline retention was ruled out, given the absence of oedemas.

In LUS, the absence of interstitial changes helped us to discard congestive causes.

#### 2.3.5 Therapeutic Intervention

Empirical antibiotic treatment was initiated due to the presence of ultrasound‐detected pulmonary infiltrates and, despite nonspecific symptoms, the low capillary oxygen saturation. Empirical high‐dose amoxicillin [21] and acetylcysteine [22, 23] were prescribed. Antihypertensive therapy was later adjusted to losartan/hydrochlorothiazide.

#### 2.3.6 Follow‐Up and Outcomes

Blood pressure achieved target values. Lung ultrasound showed gradual improvement, although residual infiltrates persisted at follow‐up.

## 3 Discussion

This case series suggests a temporal association between hypertensive urgency and subclinical pulmonary infiltrates. In all three patients, hypertensive crises occurred despite chronic antihypertensive treatment and in the absence of target organ damage or identifiable secondary causes. Lung ultrasound consistently identified pulmonary infiltrates that were not clinically apparent and, in some cases, were not detected by chest radiography. In all three cases, no congestive heart failure or hydrosaline retention of interstitial syndrome features were identified in LUS as possible differential diagnoses.

Previous evidence linked impaired respiratory function to increased blood pressure variability [24], a reduced FEV1 or vital capacity to sympathetic activation [25], and respiratory infections to endothelial dysfunction, systemic inflammation, and an increased cardiovascular risk [9, 10, 11]. Few studies have described an association between hypertensive crises and synchronous intraparenchymal pulmonary infiltrates. This is one of the first case series to describe LUS–detected subclinical pulmonary infiltrates of uncertain etiology associated with hypertensive urgency in the absence of respiratory symptoms or radiographic abnormalities.

The temporal association between pulmonary infiltrates and blood pressure elevation, followed by an improvement after empirical antibiotic therapy, suggests that subclinical inflammatory processes may be associated with hypertensive crises. However, the absence of microbiological or inflammatory confirmation prevents a causal inference.

In patients presenting with unexplained hypertensive urgencies, especially without clear triggering factors, clinicians may consider evaluating for occult inflammatory conditions, including pulmonary infiltrates detectable by lung ultrasound.

In addition to the lack of inflammatory markers and microbiological confirmation, a small sample size is another limitation of this report.

Nevertheless, the consistency of findings across cases supports further investigation of this potential association.

## 4 Conclusion

In these cases, hypertensive crises were associated with lung ultrasound‐detected pulmonary infiltrates of unknown etiology in the absence of respiratory symptoms. Although a causal relationship cannot be established, the findings suggest that subclinical inflammatory processes may contribute to blood pressure elevation. Lung ultrasound may serve as a useful complement in patients with unexplained hypertensive urgency and may help guide targeted therapy, avoiding unnecessary escalation of antihypertensive treatment and reducing associated complications. Further prospective studies incorporating microbiological and inflammatory markers are needed to clarify this potential association.

## Author Contributions

**Mónica Contreras‐Moreira:** conceptualization, data curation, formal analysis, funding acquisition, investigation, methodology, writing – original draft, writing – review and editing.

## Funding

No institutional funding was available to support the publication fees for this manuscript.

## Ethics Statement

The author declares that written informed consent was obtained from all patients for the publication of this manuscript and accompanying images, and attests that the consent form complies with journal requirements as outlined in the author guidelines.

## Conflicts of Interest

The author declares no conflicts of interest.

## Data Availability

The data that supports the findings of this study are available in Table 1 in the images attached to the corresponding cases.
